# Students’ adaptive deep learning path and teaching strategy of contemporary ceramic art under the background of Internet +

**DOI:** 10.3389/fpsyg.2022.938840

**Published:** 2022-09-02

**Authors:** Rui Zhang, Xianjing Yao, Lele Ye, Min Chen

**Affiliations:** ^1^School of Art and Design, Xinyang Normal University, Xinyang, China; ^2^College of Cultural Relics and Art, Hebei Oriental University, Langfang, China; ^3^Zhijiang College of Zhejiang University of Technology, Shaoxing, China; ^4^School of Business, Wenzhou University, Wenzhou, China

**Keywords:** Internet +, ceramic art, deep learning, teaching strategy, personalized learning

## Abstract

With the rapid expansion of Internet technology, this research aims to explore the teaching strategies of ceramic art for contemporary students. Based on deep learning (DL), an automatic question answering (QA) system is established, new teaching strategies are analyzed, and the Internet is combined with the automatic QA system to help students solve problems encountered in the process of learning. Firstly, the related theories of DL and personalized learning are analyzed. Among DL-related theories, Back Propagation Neural Network (BPNN), Convolutional Neural Network (CNN), Long Short-Term Memory (LSTM), and Gated Recurrent Unit (GRU) are compared to implement a single model and a mixed model. Secondly, the collected student questions are selected and processed, and experimental parameters in different models are set for comparative experiments. Experiments reveal that the average accuracy and Mean Reciprocal Rank (MRR) of traditional retrieval methods can only reach about 0.5. In the basic neural network, the average accuracy of LSTM and GRU structural models is about 0.81, which can achieve better results. Finally, the accuracy of the hybrid model can reach about 0.82, and the accuracy and MRR of the Bidirectional Gated Recurrent Unit Network-Attention (BiGRU-Attention) model are 0.87 and 0.89, respectively, achieving the best results. The established DL model meets the requirements of the online automatic QA system, improves the teaching system, and helps students better understand and solve problems in the ceramic art courses.

## Introduction

With the rapid development of the Internet, there is more and more integration of different fields with the Internet. Ceramic art has a profound cultural background in China, and most of the ceramic art courses in schools are about related knowledge and skills. Teachers unilaterally instill knowledge to students, which is difficult to mobilize students’ enthusiasm and learning autonomy (Zdorovets et al., 2020). The combination of the Internet and education can make use of the advantages of the Internet, establish a personalized interactive platform, enhance teaching interaction, and analyze student problems from the students’ learning situation and student psychology (Safdar et al., 2020). To help students solve learning problems in time, improve learning quality and efficiency, and help teachers explore new teaching strategies (Chang et al., 2021), an automatic question answering (QA) system is established based on deep learning (DL) by combining the Internet with an automatic QA system to improve teaching effects.

A large number of studies have been conducted on the exploration of teaching strategies in domestic and overseas. Arnesen et al. (2019) created an online module based on blended learning pedagogy, which includes a personalized learning experience; in the part of participating in a personalized course, pre-service teachers are more willing to participate in personalized learning at the end of the course than at the beginning. As teachers learn and experience personalized learning in the curriculum, teachers’ attitudes generally become more positive and confident and they pay more attention to the role of individualization in student growth (Arnesen et al., 2019). Alamri et al. (2020) used the self-determination theory as a framework to investigate the relationship between students’ perceptions of the satisfaction of their psychological needs (such as ability, autonomy, and relevance) and their intrinsic motivation when participating online courses that implement individualized learning principles. The research results indicated that the implementation of personalized learning principles in online courses can help support students’ psychological needs (such as autonomy and ability) and intrinsic motivation. In addition, students believed that personalized learning interventions were attractive and effective in meeting their learning needs and interests (Alamri et al., 2020). Hussain et al. (2020) adopted a student-centered approach and implemented a “flipped classroom” model in engineering courses based on constructivism (that is, experience-based learning) and student personalized learning. The performance of the flipped classroom teaching method and the traditional teaching method was compared, using four lenses: students’ performance, three investigations of students at different stages of the semester, teacher’s observation, and peer’s observation (Hussain et al., 2020). Al-Razgan and Alotaibi (2019) designed two stages: the first stage was a focus group consisting of five language teachers to conduct a needs analysis. The next step is to analyze the data collected in the previous stage and apply it to the system design. It consists of two parts: one is the practice part, where learners can practice specific spelling rules, and the other is the game part, where learners can play spelling games and score points (Al-Razgan and Alotaibi, 2019). Xu (2021) proposed that the educational nature of multimedia technology is its fundamental attribute. The application of multimedia technology in the field of education is often carried out in combination with the content of the syllabus, teaching objectives, and goals. It is to use the audition function of multimedia in specific classroom teaching activities to transmit relevant knowledge to students in a more vivid and flexible form, which can help students better understand and master relevant knowledge and skills. The artistic characteristics of multimedia technology provide an important guarantee for the smooth development of ceramic art course teaching (Xu, 2021). Luo (2021) pointed out that ceramic art teaching is often combined with other disciplines, and it will certainly be able to shine. Combined with tea culture, ceramic art teaching can introduce the development of ceramic craftsmanship from the perspective of utensils, and the influence of glaze color and texture on ceramic art from the perspective of color. And it can also form a new esthetic meaning under the influence of tea culture esthetics such as tea ceremony, tea science, tea painting, and tea art. Combined with art, it can introduce the composition and emotional expression of ceramic art from the perspective of the similarities and differences between ancient and modern Chinese and foreign art. Combined with history, the characteristics of famous kilns of various generations can be introduced from the perspective of unearthed cultural relics. Combined with software programming, from the perspective of mobile games of ceramic art, the basic process and core keys of completing ceramic art can be introduced. By allowing students to dabble in different disciplines, it is beneficial for students to improve their knowledge system, broaden their horizons, and meanwhile, they can understand the history of ceramic art development and build a theoretical system of ceramic art (Luo, 2021).

Internet + education can make up for the shortcomings of the traditional teaching model, so that students can actively learn and consolidate knowledge and check for omissions according to their own learning situation in spare time. Internet + education can also analyze the psychological related problems of students according to their current status in terms of students’ learning psychological problems. In terms of knowledge learning, when students encounter a problem, they often use commercial engines to search for answers, but it is often difficult to solve the problem quickly and accurately, which leads to the accumulation of problems and affects the learning effect (Urokova, 2020). The establishment of an automatic QA system based on the DL network, as a supplement to the new teaching model, can help students get help faster and more accurately, and enhance their interest in learning.

## Personalized Q&A system for students in the context of Internet +

### DL-related theories

Deep learning is a new research direction in the field of Machine Learning (ML). DL is the inherent law and representation level of sample data. The information obtained in the learning process is of great help to the interpretation of data such as text, images, and sounds (Wang et al., 2020). The ultimate goal of DL is to enable machines to have human-like learning and analysis capabilities. It is a complex ML algorithm that far surpasses previous related technologies in speech and image recognition (Pang et al., 2021). Most ML algorithms use supervised learning. A supervised learning algorithm is an algorithm, in which any type of the variable must be input and produce an output variable. Its purpose is to approximate the mapping function to a level where it is possible to have new input data that consistently predict appropriate values for that particular data. In an unsupervised ML algorithm, only the input data are required and no output variables need to be provided. Its main purpose is to model the underlying structure or distribution of data to obtain more data. Semi-supervised algorithms are where most real-world problems lie, and the se types of algorithms fall somewhere between supervised and unsupervised algorithms.

**Figure 1:**
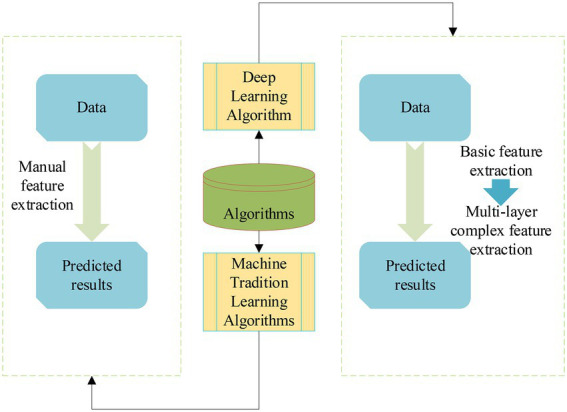
Flow chart of DL and ML algorithms.

The flow of the DL algorithm and ML algorithm is displayed in Figure 1.

**Figure 2:**
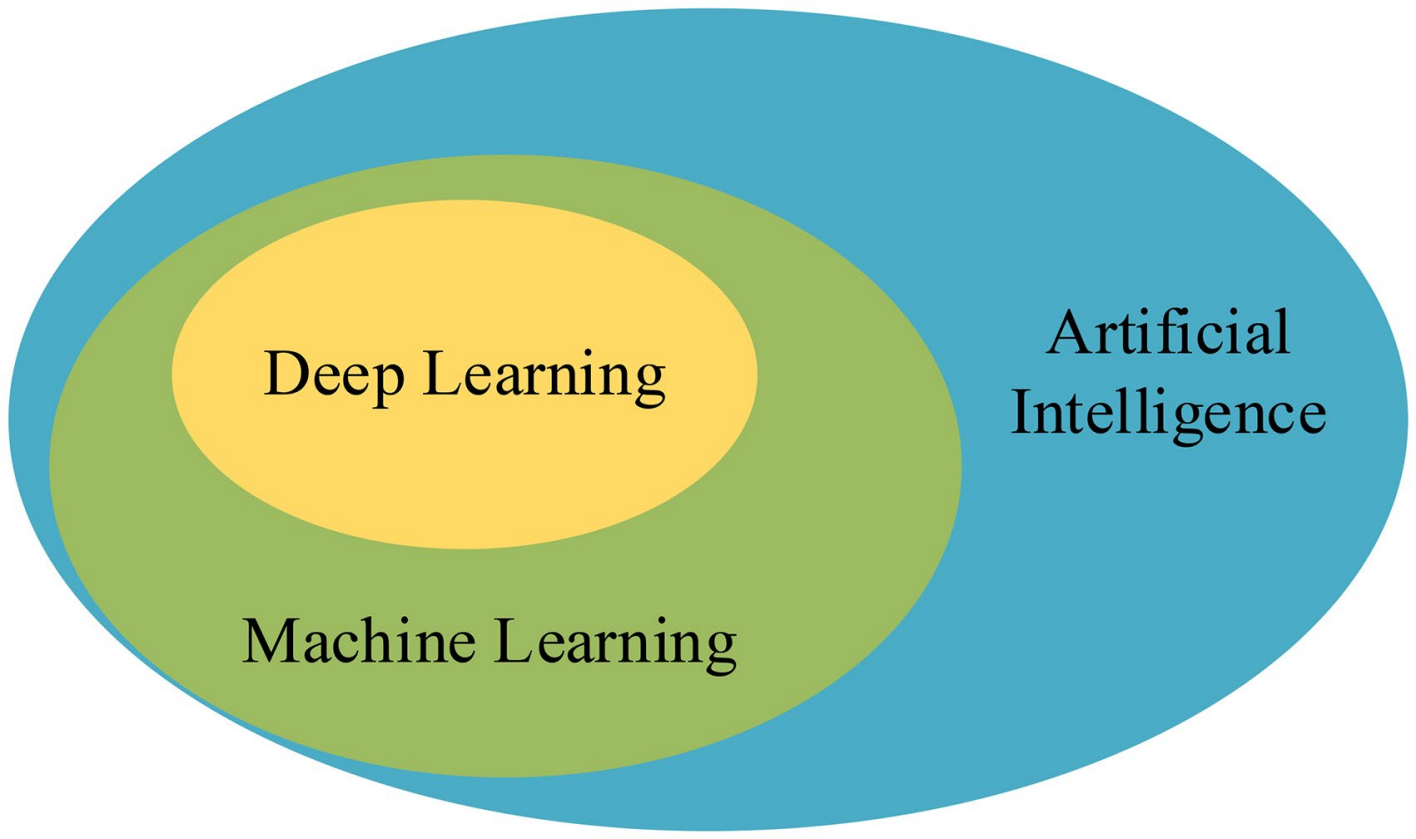
The relationship between AI, ML, and DL.

Deep learning algorithms have more advantages than traditional ML algorithms in implementing models and extracting feature data. Many problems in the development of artificial intelligence (AI) have been effectively solved with the development of DL. AI is to endow machines with human intelligence, so that computers have the ability to judge some situations like people; ML is to use algorithms to analyze data to achieve specific tasks, which is a method to achieve AI (Zhu et al., 2020). DL optimizes the early algorithms of ML and is a better technique, and their relationship is exhibited in Figure 2.

### Individualized learning theory

Personalized learning is to discover and solve the learning problems of students through the comprehensive evaluation of specific students, and tailor learning strategies and learning methods different from others for students. Every student is unique, with his unique talents, strengths, and weaknesses. Individualized methods should be used to solve children’s learning problems (Tsai et al., 2020). The idea of personalized learning has also been mentioned by more and more experts. With the rapid development of information technology, a variety of new technologies have also motivated solving the problem of personalized learning (Kurilovas, 2019). In addition to curriculum teaching design, student learning has other factors that affect academic performance, such as students’ gender, parental support, learning concepts, and learning orientation (Hwang and Fu, 2020). Some scholars have studied other factors that affect students’ academic achievement, including learning orientation, learning concepts, learning motivation, academic commitment, and parental support. According to different backgrounds and environments, it needs to study and analyze internal and external factors separately about students’ learning (Erwiza et al., 2019).

In summary, scholars’ research on personalized learning has gradually shifted from focusing on learning content and learning navigation to analyzing educational psychological factors such as learning styles. Data analysis of personalized learning requires the design of accurate personalized learning services for students. (McCarthy et al., 2020). Personalized learning is already one of the basic characteristics of current learning and a new direction of current education and learning.

### Back propagation neural network

**Figure 3:**
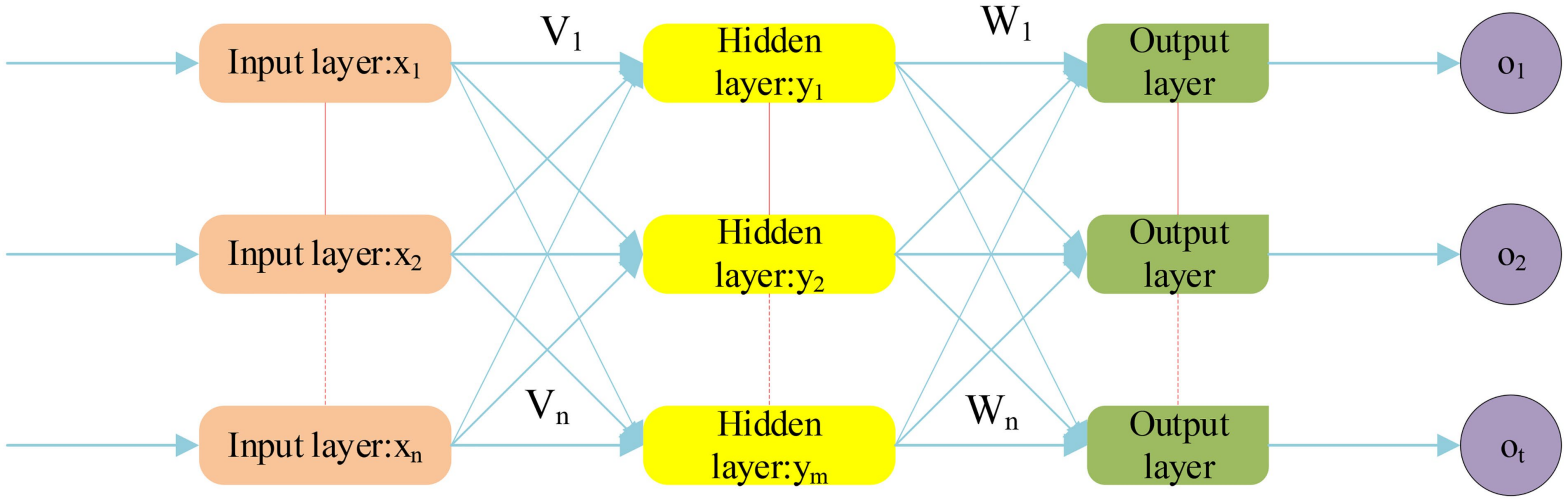
Forward propagation stage of BPNN structure.

Back Propagation Neural Network (BPNN) mainly includes two stages, namely forward propagation and backpropagation. The forward propagation stage refers to a process in which the excitation signal is first introduced from the input layer, then processed by the hidden layer, and finally to the output layer (Ruan et al., 2020). If the output of the output layer is inconsistent with the actual output, it will enter another stage of backpropagation. The backpropagation stage is one of the cores of the BPNN model. The operating principle is to propagate the wrong information to the input layer through the hidden layer through its own characteristics. But in actual work to adjust it, the purpose is to output the correct value. Therefore, it mainly shows the forward propagation stage in the BPNN model, as expressed in Figure 3.

The input is a n-dimensional feature vector: $X={\left(x_{1},x_{2},\ldots ,x_{n}\right)}^{T}$, and the output is a m-dimensional feature vector: ${\left(y_{1},y_{2},\ldots ,y_{m}\right)}^{T}$. V means the weight between the input layer and the hidden layer, and W means the weight between the hidden layer and the output layer. The training process of BPNN is mainly divided into two parts, signal forward propagation and error information back feedback. The Sigmoid function is used in the hidden layer, and the equation is as follows.


(1) $$f\left(a\right)=\frac{1}{1+e^{-x}}$$

The BPNN algorithm is divided into the following steps. First, initialize the network, take random numbers in (−1, 1) to initialize (weight) and b (threshold). M means a maximum number of training times, ε means calculation accuracy, and e means the error function. The k-th input sample and expected output:


(2) $$\begin{array}{c} x\left(k\right)=\left(x_{1}\left(k\right),\;x_{2}\left(k\right),\ldots ,\;x_{n}\left(k\right)\right)\\ d_{o}\left(k\right)=\left(d_{1}\left(k\right),\;d_{2}\left(k\right),\ldots ,\;d_{n}\left(k\right)\right) \end{array}$$

The input and output of each neuron in the hidden layer and output layer:


(3) $$\begin{array}{cc} hi_{h}\left(k\right)=\sum_{i=1}^{n}w_{ih}x_{i}\left(k\right)-b_{h} & h=\left(1,2\ldots ,\;p\right)\\ ho_{h}\left(k\right)=f\left(hi_{h}\left(k\right)\right) & h=\left(1,2\ldots ,\;p\right)\\ \begin{array}{c} {yi}_{n}\left(k\right)=\sum_{h=1}^{p}w_{ho}ho_{h}\left(k\right)-b_{o}\\ {yo}_{o}\left(k\right)=f\left({yi}_{o}\left(k\right)\right) \end{array} & \begin{array}{c} o=\left(1,2\ldots ,\;p\right)\\ o=\left(1,2\ldots ,\;p\right) \end{array} \end{array}$$

Define the error function as:


(4) $$\begin{array}{c} e=\frac{1}{2}\sum_{o=1}^{q}{\left(d_{0}\left(k\right)-{yo}_{o}\left(k\right)\right)}^{2}\\ \frac{\partial e}{\partial w_{ho}}=\frac{\partial e}{\partial yi_{o}}\frac{\partial yi_{o}}{\partial w_{ho}}\\ \frac{\partial yi_{o}\left(k\right)}{\partial w_{ho}}=\frac{\partial \left(\sum_{h}^{p}w_{ho}ho_{h}\left(k\right)-b_{o}\right)}{\partial w_{ho}}=ho_{h}\left(k\right)\\ \frac{\partial e}{\partial yi_{o}}=\frac{\partial {\left(\frac{1}{2}\sum_{o=1}^{q}\left(d_{o}\left(k\right)-{yo}_{o}\left(k\right)\right)\right)}^{2}}{\partial yi_{o}}\\ =\partial \left(d_{o}\left(k\right)-{yo}_{o}\left(k\right)\right){yo}_{o}\left(k\right)\\ =-\left(d_{o}\left(k\right)-{yo}_{o}\left(k\right)\right)f\left({yi}_{o}\left(k\right)\right)=\delta_{o}\left(k\right) \end{array}$$

The error function can be used to measure the training result. The smaller the error function value is better, so the weight value is modified to reduce the error function value. The equation is as follows:


(5) $$\begin{array}{c} \frac{\partial e}{\partial hi_{h}\left(k\right)}=\frac{\partial \left(\frac{1}{2}\sum_{o=1}^{q}{\left(d_{o}\left(k\right)-{yo}_{o}\left(k\right)\right)}^{2}\right)}{\partial h0_{h}\left(k\right)}\frac{\partial ho_{h}\left(k\right)}{\partial hi_{h}\left(k\right)}\\ =\frac{\partial \left(\frac{1}{2}\sum_{o=q}^{q}{\left(d_{o}\left(k\right)-f\left({yi}_{o}\left(k\right)\right)\right)}^{2}\right)}{\partial ho_{h}\left(k\right)}\frac{\partial ho_{h}\left(k\right)}{\partial hi_{h}\left(k\right)}\\ =\frac{\partial \left(\frac{1}{2}\sum_{n=1}^{q}\left(d_{o}\left(k\right)-f{\left(\sum_{h=1}^{p}w_{ho}ho_{h}\left(k\right)-b_{o}\right)}^{2}\right)\right)}{\partial ho_{h}\left(k\right)}\frac{\partial ho_{h}\left(k\right)}{\partial hi_{h}\left(k\right)}\\ =-\sum_{o=1}^{q}\left(d_{o}\left(k\right)-{yo}_{o}\left(k\right)\right)f^{\prime }\left({yi}_{o}\left(k\right)\right)w_{ho}\frac{\partial ho_{h}\left(k\right)}{\partial hi_{h}\left(k\right)}\\ =-\left(\sum_{o=1}^{q}\left(d_{o}\left(k\right)w_{ho})f^{\prime }(hi_{h}\left(k\right)\right)\right)\\ =\delta_{h}\left(k\right) \end{array}$$

Then, update the connection weight $w_{ho}$:


(6) $$\begin{array}{c} \Delta w_{ho}\left(k\right)=-\eta \frac{\partial e}{\partial w_{ho}}=\eta \delta_{o}\left(k\right)ho_{h}\left(k\right)\\ w_{ho}^{N+1}=w_{ho}^{N}+\eta \delta_{o}\left(k\right)ho_{h}\left(k\right) \end{array}$$

η means learning rate and $w_{ho}$ means connection weight. The threshold change is obtained as Equation (7). Finally, the global error is calculated as in Equation (7).


(8) $$\begin{array}{c} \Delta b_{o}\left(k\right)=\eta *\delta_{o}\left(k\right)\\ \Delta b_{h}\left(k\right)=\eta *\delta_{h}\left(k\right) \end{array}$$

(8) $$E=\frac{1}{2m}\sum_{k=1}^{m}\sum_{o=1}^{q}{\left(d_{o}\left(k\right)-y_{o}\left(k\right)\right)}^{2}$$

**Figure 4:**
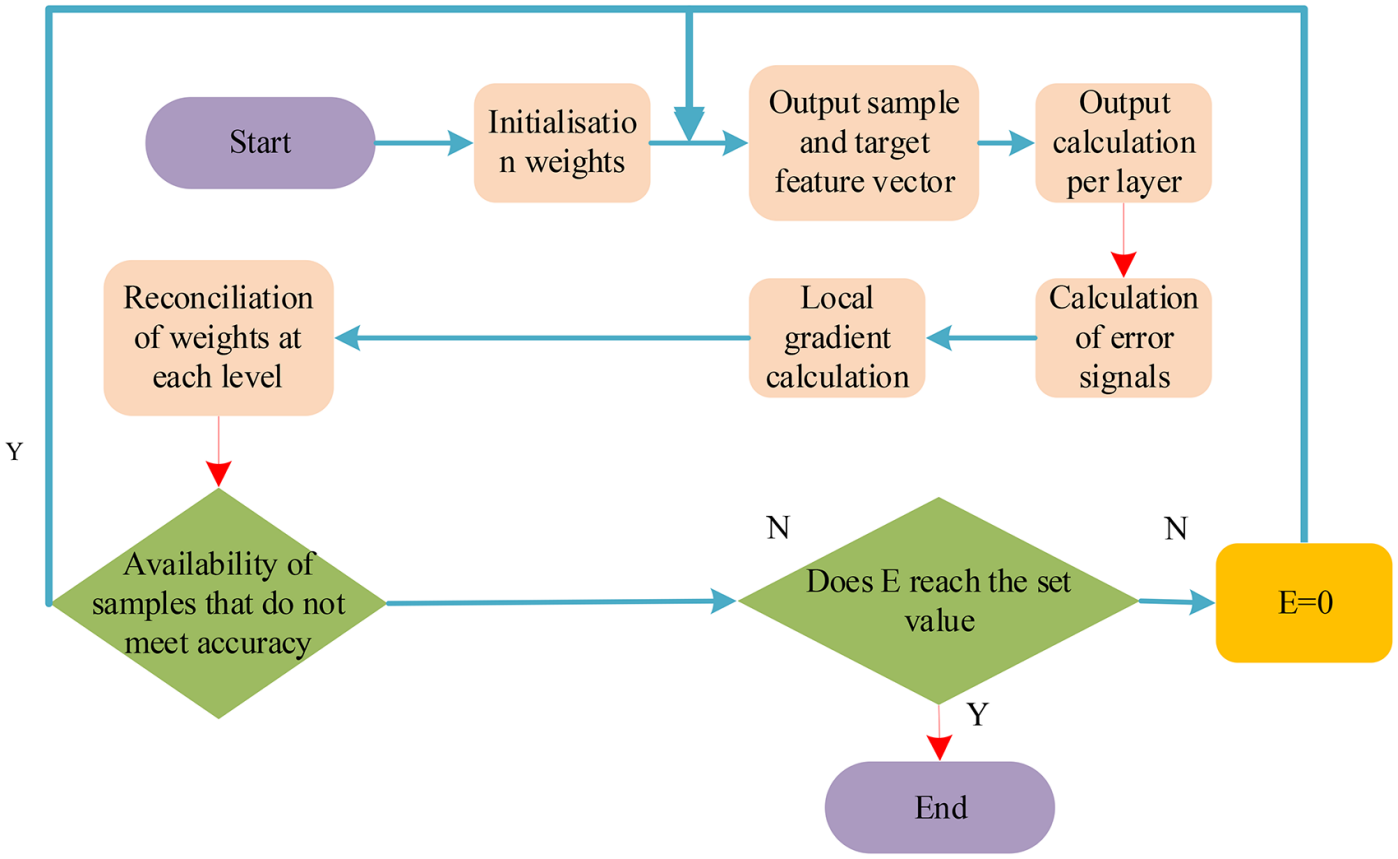
Flow chart of BP algorithm.

If the final prediction error of the neural network (NN) reaches the preset value or reaches the maximum number of training times, it will stop training. If it does not meet the requirements, the next sample is input for the next round of learning and training with the expected output value. Figure 4 shows the BP algorithm flow.

## Research model

### Convolutional neural network

**Figure 5:**
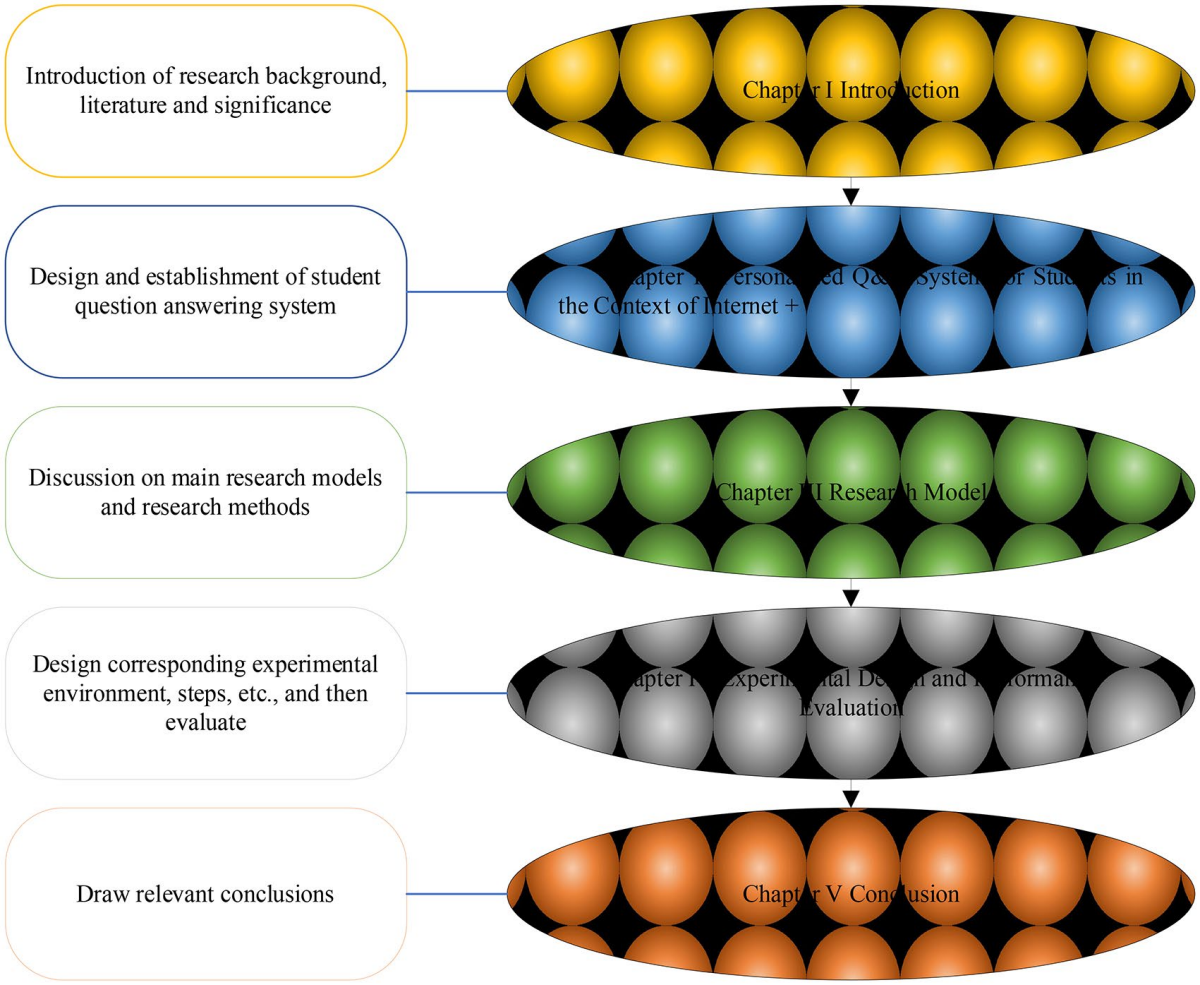
System architecture.

Under the background of the Internet, the exploration of students’ adaptive DL path of contemporary ceramic art and the analysis of teaching strategies are carried out. The specific system architecture is expressed in Figure 5.

**Figure 6:**
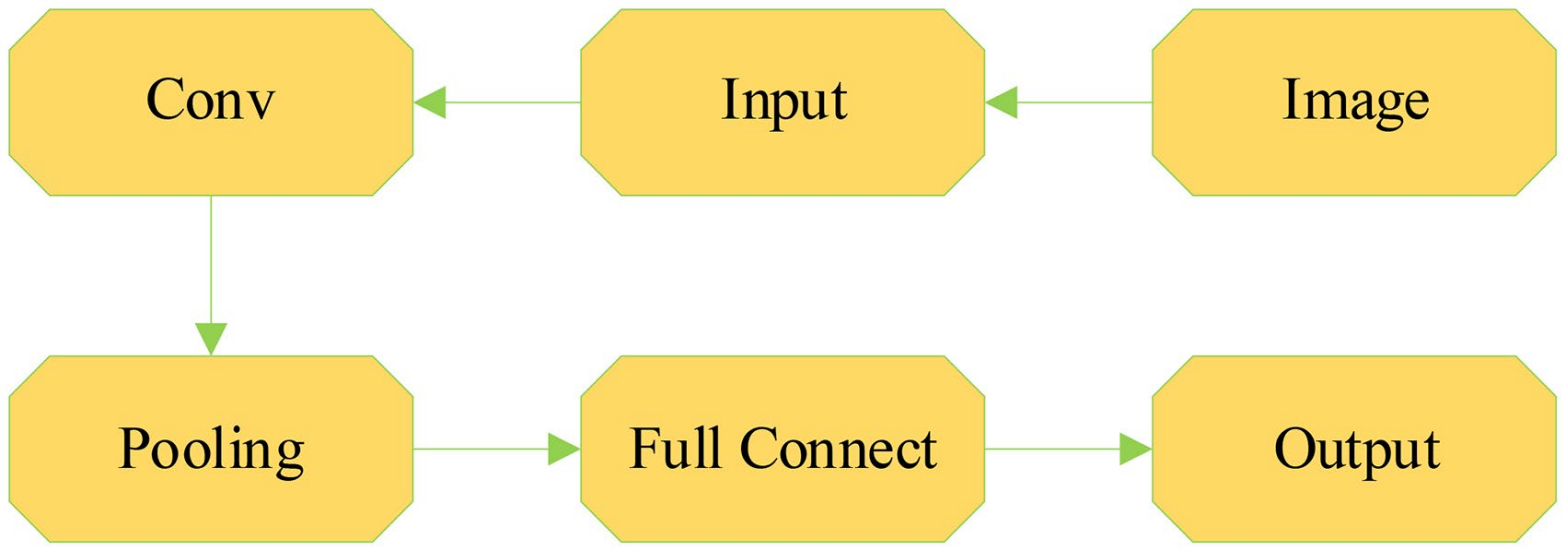
Frame diagram of CNN.

The main advantage of CNN is that increasing the number of network layers can model complex non-linear relationships (Kattenborn et al., 2021). CNN includes the input layer, one or more convolutional layers, the output layer, the pooling layer, and the dropout. Figure 6 is a simplified structure diagram of CNN.

The basic two-dimensional convolution is as in Equation (9), where x means the sample matrix, size of filter w is represented by U*V, and output y means convolution of signal sequence x and filter w.


(9) $$y_{i,j}=\sum_{u=1}^{U}\sum_{v=1}^{v}w_{u,v}x_{i-u+1,j-v+1}$$

Assuming that the output sample y starting from (3,3), $y_{3,5}=\sum_{u=1}^{3}\sum_{v=1}^{3}w_{u,v}x_{3-u+1,3-v+1}=-1$ can be gained, then the output matrix can be obtained. The continuous and discrete forms are as follows.


(10) $$\begin{array}{c} y\left(n\right)=\left(f⊗w\right)\left(n\right)=\int_{-\infty }^{\infty }f\left(x\right)w\left(n-x\right)dx\\ y\left(n\right)=\left(f⊗w\right)\left(n\right)=\sum_{x=-\infty }^{\infty }f\left(x\right)w\left(n-x\right) \end{array}$$

⊗ means convolution operation, *f* means input function, *w* represents weighting function, *y* means feature map, *x* represents a map, and *w* means filter. Input the result of feature mapping into the next layer as follows.


(11) $$\begin{array}{c} \;z^{l}=w^{l}⊗x+b^{l}\\ y^{l}=f\left(z^{l}\right) \end{array}$$

Characteristics of convolutional layer: Local connections are shared with convolution kernels. The purpose of the local connection is to reduce the number of parameters, increase the calculation speed, and effectively reduce the probability of overfitting. Convolution kernel sharing means that when extracting feature maps, the same convolution kernel is shared between locations, which is also to reduce the number of parameters and further increase the calculation speed. The pooling layer mainly compresses data, reduces parameters, and improves calculation speed. Fully connected layer: It is the hidden layer of the traditional NN. Each neuron in this layer needs to be connected to the previous neuron. In CNN, the convolutional layer, pooling layer, and fully connected layer need to add activation functions. The activation function is to activate neurons to reduce the probability of overfitting. Commonly used activation functions mainly include Sigmoid-type functions and ReLU functions. Among them, the commonly used forms of Sigmoid-type functions are Logistic function and Tanh function, as shown in the following equation:


(12) $$a=f\left(z^{l}\right)$$

(13) $$\begin{array}{c} \sigma \left(x\right)=\frac{1}{1+e^{-x}}\\ \mathrm{Tanh}-\left(x\right)=\frac{e^{x}-e^{-x}}{e^{x}+e^{-x}} \end{array}$$

(14) $$\mathrm{ReLU}\left(x\right)=\{\begin{array}{cc} x & x\geq 0\\ 0 & x<0 \end{array}$$

**Figure 7:**
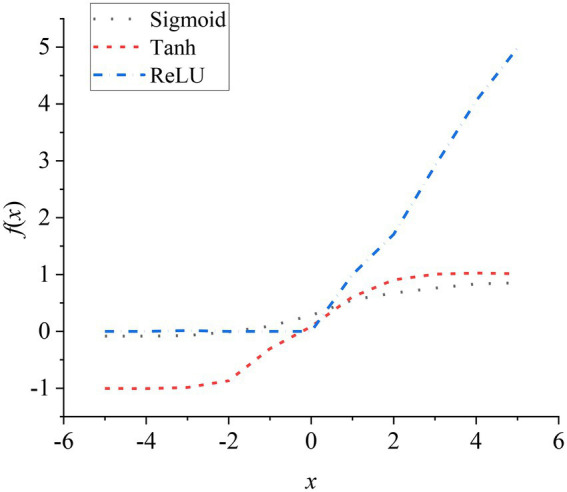
Sigmoid, Tanh, and ReLU activation function curves.

Figure 7 is a graph of Sigmoid, Tanh, and ReLU functions.

Tanh function: The output value of the function is centered at 0. Although it has a faster convergence speed, there is a gradient dispersion. ReLU function is currently a commonly used activation function, which solves the gradient dispersion. And it has a fast convergence speed and it can reduce the possibility of overfitting. The LeakyReLU activation function is an improved version of the ReLU function. The calculation equation is as follows, a generally takes 0.01 as a constant value.


(15) $$f\left(x\right)=\{\begin{array}{cc} x, & x\geq 0\\ \mathrm{ax}, & x<0 \end{array}$$

The weight update in the NN is generally the BP algorithm, and CNN also uses the BP algorithm to update the weight and propagate forward. The output of the i-layer is as the following equation:


(16) $$x^{i}=f\left(u^{i}\right)$$

(17) $$u^{i}=W^{i}x^{i-1}+b^{i}$$

W means i-layer weight and b represents i-layer bias. Suppose the overall loss function of the NN:


(18) $$E^{N}=\frac{1}{2}\sum_{n=1}^{N}\sum_{k=1}^{c}{\left(t_{k}^{n}-y_{k}^{n}\right)}^{2}$$

*N* means the total number of samples and *c* means the number of sample categories. $t_{k}^{n}$ represents the nth sample label of the k-th dimension, and $y_{k}^{n}$ represents the output of the n-th sample of the k-th dimension. Each layer of CNN uses the gradient descent method to update the weight, and the calculation formula for the weight update and bias update:


(19) $$W_{n}^{i}=W_{o}^{i}-\eta \frac{\partial E}{\partial W_{o}^{i}}$$

(20) $$b_{n}^{i}=b_{o}^{i}-\eta \frac{\partial E}{\partial b_{o}^{i}}$$

$W_{o}^{i}$ and $b_{o}^{i}$ represent Weight and bias before update; $W_{n}^{i}$ and $b_{n}^{i}$ represent Weight and bias after the update. ηmeans the learning rate in the gradient descent method. The convolutional layer in CNN propagates forward, and the output feature map of each i-layer convolution:


(21) $$x_{j}^{i}=f\left(\underset{l\in M_{j}}{\sum }\;x_{l}^{i-1}*k_{lj}^{i}+b_{j}\right)$$

*M_j_* means input feature map; $k_{lj}^{i}$ represents the convolution kernel, and *f* means activation function. With CNN backpropagation, pooling layer error backpropagation as Equation (22), convolutional layer error direction propagation Equation (23), convolutional layer weight update, bias update as (Equation 24 and 25).


(22) $$\delta^{i-1}=\mathrm{upsample}\left(\delta^{i}\right)⊙\sigma^{\prime }\left(u^{i-1}\right)$$

$$\delta^{i-1}=\delta^{i}\left(\partial \delta^{i}/\partial \delta^{i-1}\right)=\delta^{i}*\mathrm{rot}180\left(W^{i}\right)⊙\sigma^{\prime }\left(u^{i-1}\right)$$

(24) $$\partial E/\partial W^{i}=\left(\partial E/\partial u^{i}\right)\left(\partial u^{i}/\partial W^{i}\right)=x^{i-1}*\delta^{i}$$

(25) $$\partial E/\partial b^{i}=\sum u,v\left(\delta^{i}\right)u,v$$

$\mathrm{upsample}\left(\delta^{i}\right)$ means sampling, ⊙ represents Hadamard product, δ represents error value, *i* means *i* layers, and *E* means loss function (Bocci and Carlini, 2022).

### Long short-term memory

**Figure 8:**
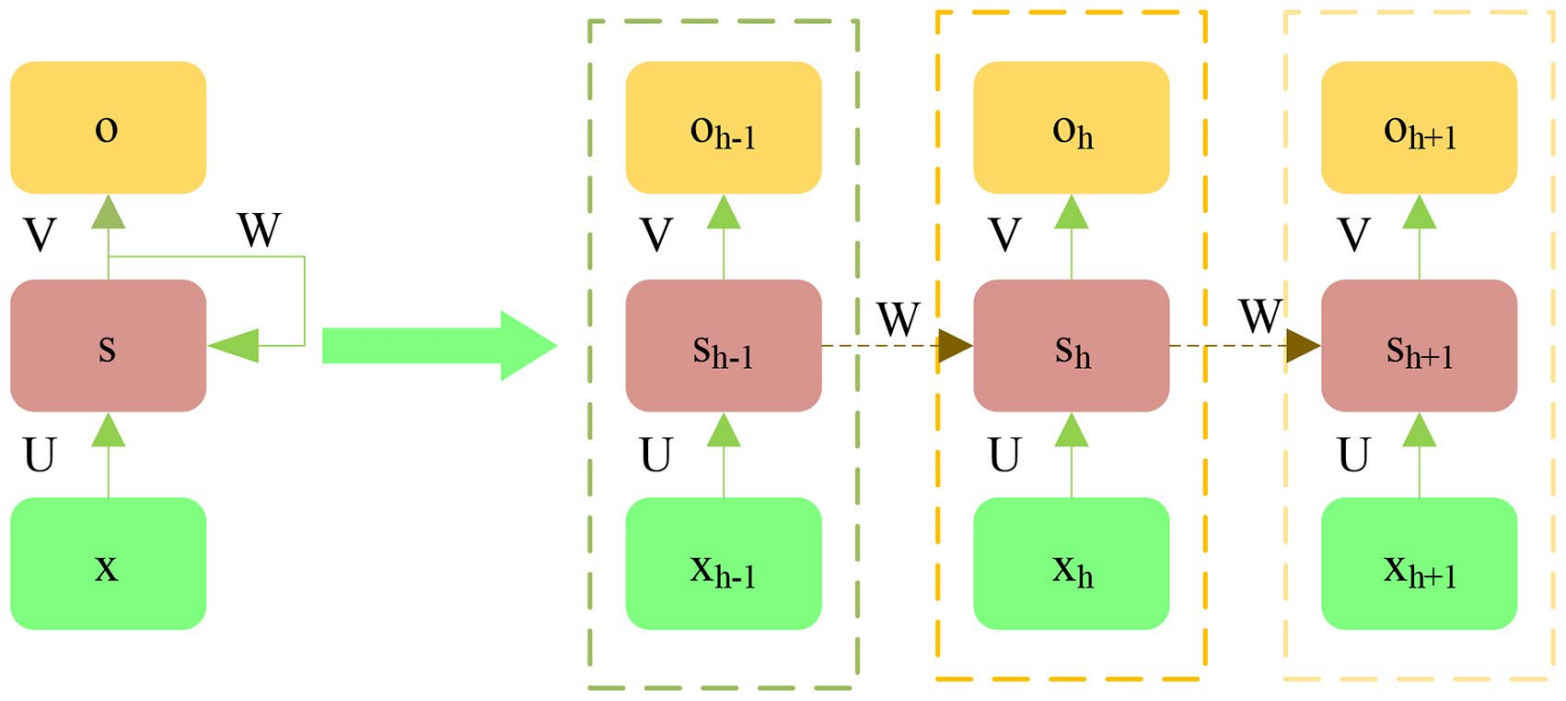
Structure diagram of RNN.

Compared with traditional NNs, Recurrent Neural Network (RNN) mainly adds time to the loop structure, so that when processing data, the processed information can retain some “memory,” and it can be free from the limit of sequence length when processing data. Figure 8 shows the structure of RNN.

x: the input at the current time h; s: hide the node status in real-time; o: output (RNN processing). The specific equation is:


(26) $$s_{t}=f\left(V_{x_{h}}+W_{s_{h-1}}+b\right)$$

(27) $$o_{t}=\mathrm{softmax}\left(V_{s_{h}}+c\right)$$

*f* represents activation function sigmoid; *V* and *W* are weight matrixes between layers; and *b* and *c*: bias value. The shortcomings of RNN are also obvious, which includes unstable model parameter update, the gradient exploding or disappearing, and the memory being short. The LSTM model is an improvement of the cyclic NN model. In the structure, the “gate” structure is added, so that the problems caused by the long distance can be solved, even if the length of the data sequence is different. LSTM model neuron is composed of the unit state, the output gate, the input gate, and the forget gate. The working model of the forget gate: The sigmoid function assigns the weighted calculated value of the input pt. at the current time *t* and the output kt-1 at the time t-1, and uses the above to control the influence of the previous output sequence information on the input stream. The equation is as (28):


(28) $$g_{t}=\sigma \left(W_{f}\cdot \left[k_{t-1},p_{t}\right]\right)+b_{g}$$

The sigmoid function is used to weight the input pt. and the output kt-1 at t-1 to obtain the value s, as shown in the following Equation (29). The new state candidate value of the unit is generated by the non-linear Tanh function, as shown in the following Equation (30). The new state At of the unit only needs to add the two, and then passes through the forget gate and the input gate, as shown in Equation (31):


(29) $$s_{t}=\sigma \left(W_{i}\cdot \left[k_{t-1},p_{t}\right]\right)+b_{s}$$

(30) $$\tilde{A_{t}}=\mathrm{tann}\left(W_{c}\cdot \left[k_{t-1},p_{t}\right]\right)+b_{A}$$

(31) $$A_{t}=g_{t}\cdot A_{t-1}+s_{t}\cdot \tilde{A_{t}}$$

The value qt output by the gate output needs to use the sigmoid function to weight the input pt. and the output $k_{t-1}$ at t-1, as shown in the following Equation (32). Next, the output of the LSTM unit is controlled by the non-linear Tanh function calculation, and finally, the output value kt is shown in the following Equation (33). The advantage of LSTM is to solve the data problem due to the long distance.


(32) $$q_{t}=\sigma \left(W_{q}\cdot \left[k_{t-1},p_{t}\right]\right)+b_{q}$$

(33) $$k_{t}=q_{t}\cdot \mathrm{tann}\left(A_{t}\right)$$

**Figure 9:**
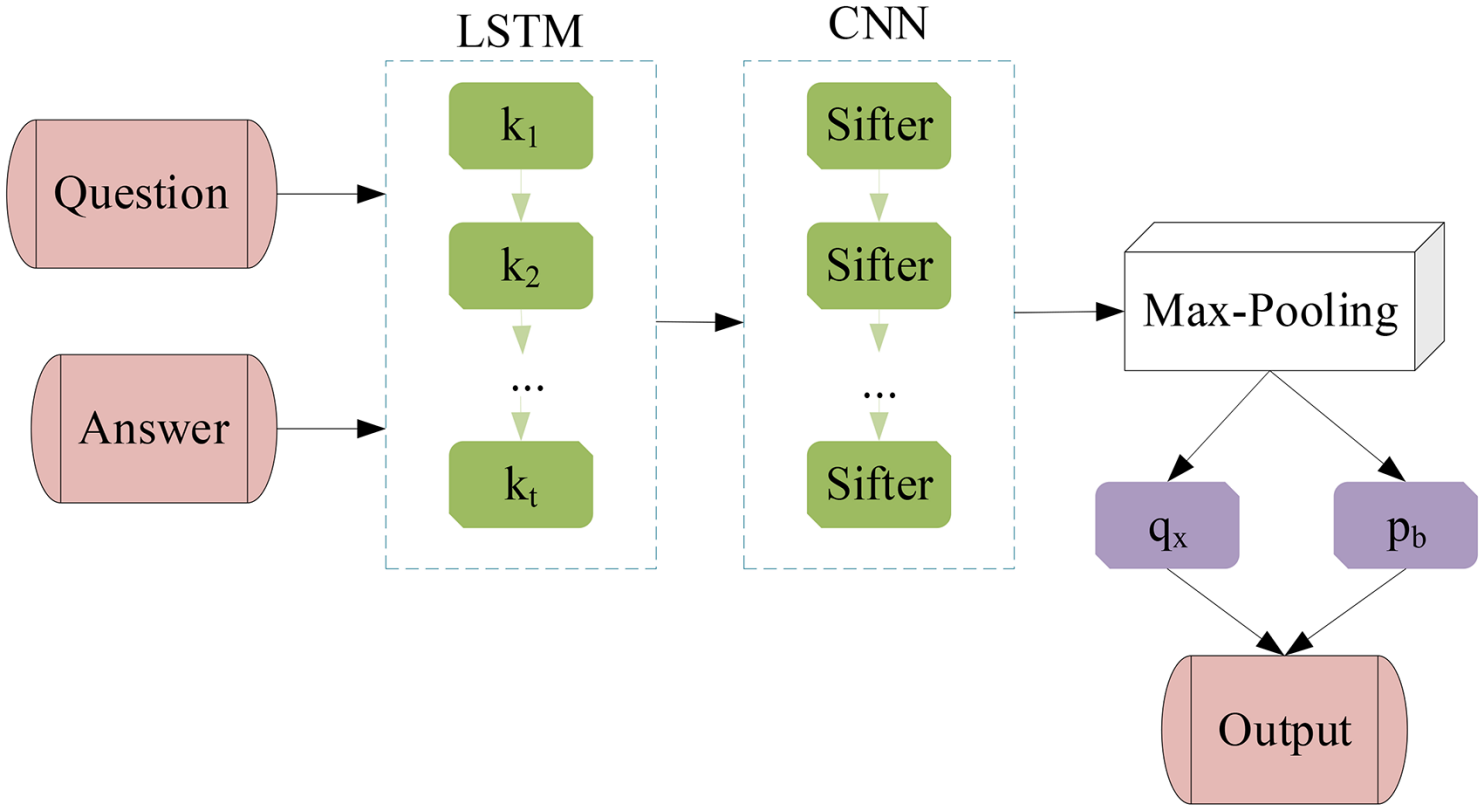
The structure diagram of LSTM-CNN model.

Through the previous introduction of different network models, the LSTM model is combined with the CNN model to obtain a hybrid network model based on the CNN model. The structure of the hybrid neural network (HNN) model is shown in Figure 9 below.

### Gated recurrent unit

Gated Recurrent Unit (GRU) is a variant of LSTM. The input gate and forget gate in LSTM are merged into update gates and some other changes to form GRU (Shahid et al., 2020). GRU has few parameters and fast convergence. Input the sequence $x=\left\{x_{1},x_{2},x_{3},\ldots x_{t}\right\}$ to the GRU, the internal state is as follows:


(34) $$z_{t}=\sigma \left(W_{z}\cdot \left[h_{t-1},x_{t}\right]\right)$$

(35) $$r_{t}=\sigma \left(W_{r}\cdot \left[h_{t-1},x_{t}\right]\right)$$

(36) $$h_{t}=\tanh\left(W_{h}\cdot \left[r_{t}*h_{t-1},x_{t}\right]\right)$$

(37) $$h_{t}=\left(1-z_{t}\right)*h_{t-1}+z_{t}*h_{t}$$

(38) $$y_{t}=\sigma \left(W_{o}\cdot h_{t}\right)$$

$x_{t}$ represents input vector; $h_{t-1}$ represents hidden layer output at the previous moment; $z_{t}$ means update gate; $r_{t}$ means reset gate; ∗ represents multiply by point; σ represents sigmoid activation function; *W* represents weight matrix; and dropout is added during training to prevent overfitting.

The Attention mechanism simulates the attention model of the human brain, which is essentially a resource allocation model. Basic working principle: reasonable allocation of attention resources, more allocations to key parts, and less allocation to the rest, which can reduce refers to eliminating the adverse effects of non-key parts. Commonly used methods are used to score functions for soft attention and experiment with three weight value calculations. The first is to input all attention models to score and sum, as in Equation (39):


(39) $$\alpha_{t}=\frac{\exp\left(\mathrm{score}\left(h_{t}\right)\right)}{\sum_{i=1}^{T}\exp\left(\mathrm{score}\left(h_{t}\right)\right)}$$

$\alpha_{t}$: The weight value of the t-th input; $h_{t}$: the t-th input; score(): the score of the input. The second type is to calculate the input first, and then the calculated input model, such as Equation (40):


(40) $$y_{t}=\tan h\left(Wh_{t}\right)$$

(41) $$\alpha_{t}=\frac{\exp\left(\mathrm{score}\left(y_{t}\right)\right)}{\sum_{i=1}^{T}\exp\left(\mathrm{score}\left(y_{t}\right)\right)}$$

$y_{t}$ is gained by calculating $h_{t}$; W: the output obtained by inputting the single-layer NN;$\mathrm{and}\;\alpha_{t}$is the input weight value. The first two methods are after obtaining$\alpha_{t}$, multiply $\alpha_{t}$and $h_{t}$, and finally accumulate, as in Equation (42):


(42) $$S_{t}=\sum_{i=0}^{n}\alpha_{t}h_{t}$$

St: the output of the input model. The third type is obtained based on the second type, and its own input for calculation, such as Equation (43):


(43) $$S_{t}=\sum_{i=0}^{n}\left(\alpha_{t}h_{t}+h_{t}\right)$$

**Figure 10:**
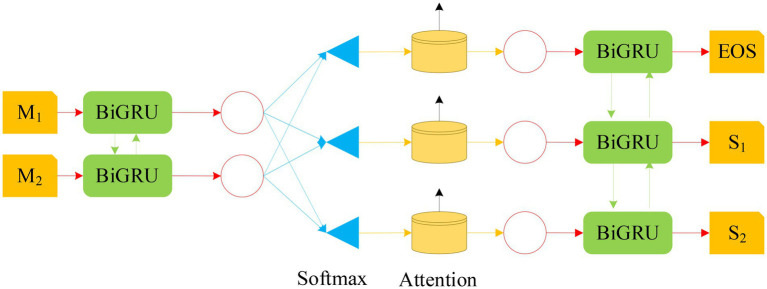
The structure diagram of BiGRU-Attention model (M1 and M2 in the figure represent input random variables; S1 and S2 stand for output random variables).

According to the GRU and Attention mechanism, a NN model based on BiGRU-Attention (Bidirectional Gated Recurrent Unit Network-Attention) is constructed, as shown in Figure 10.

## Experimental design and performance evaluation

### Experimental environment and data processing

**Figure 11:**
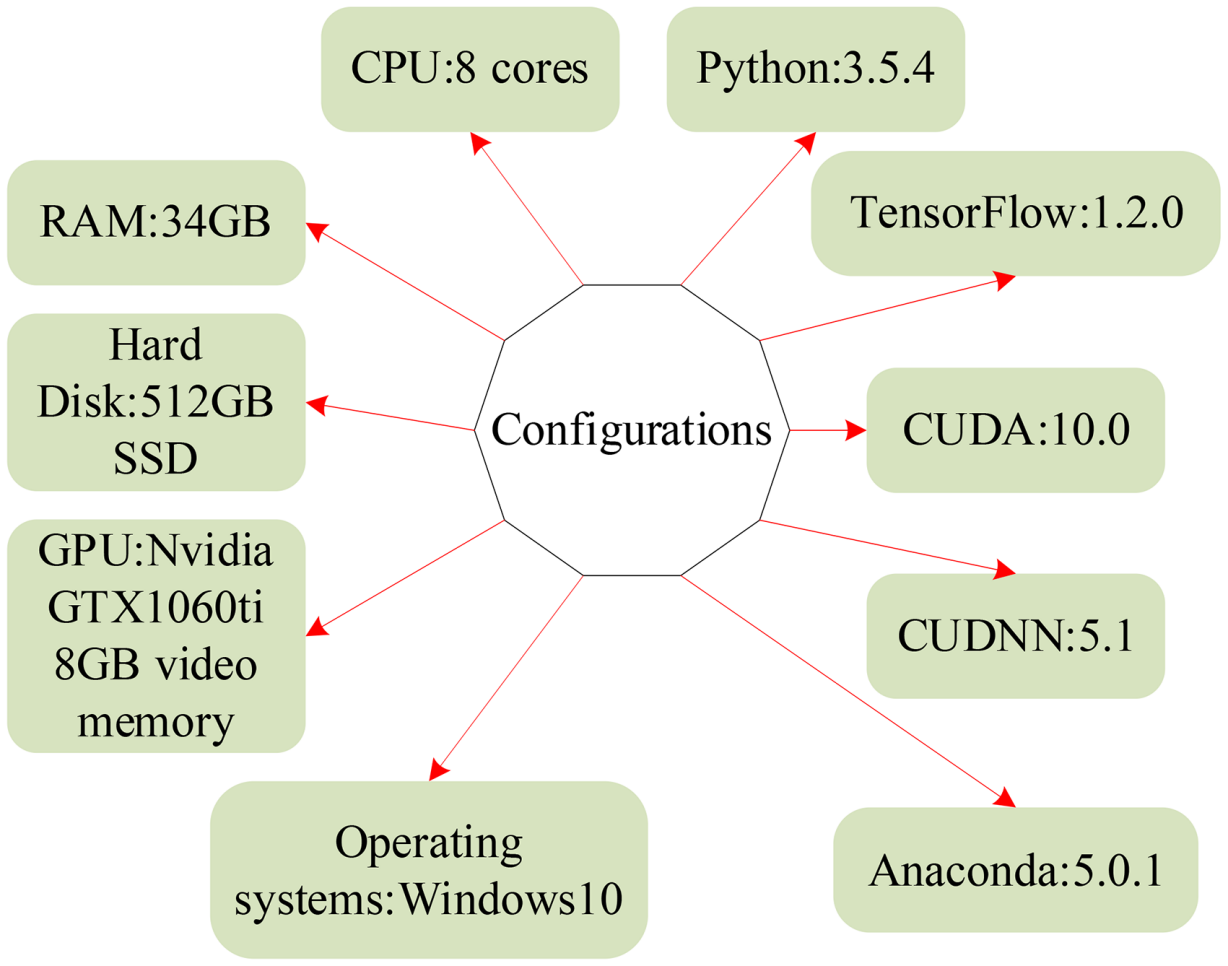
Debugging environment and coding platform.

There are two experimental environments, as shown in Figure 11 for the platform for writing and debugging code.

**Figure 12:**
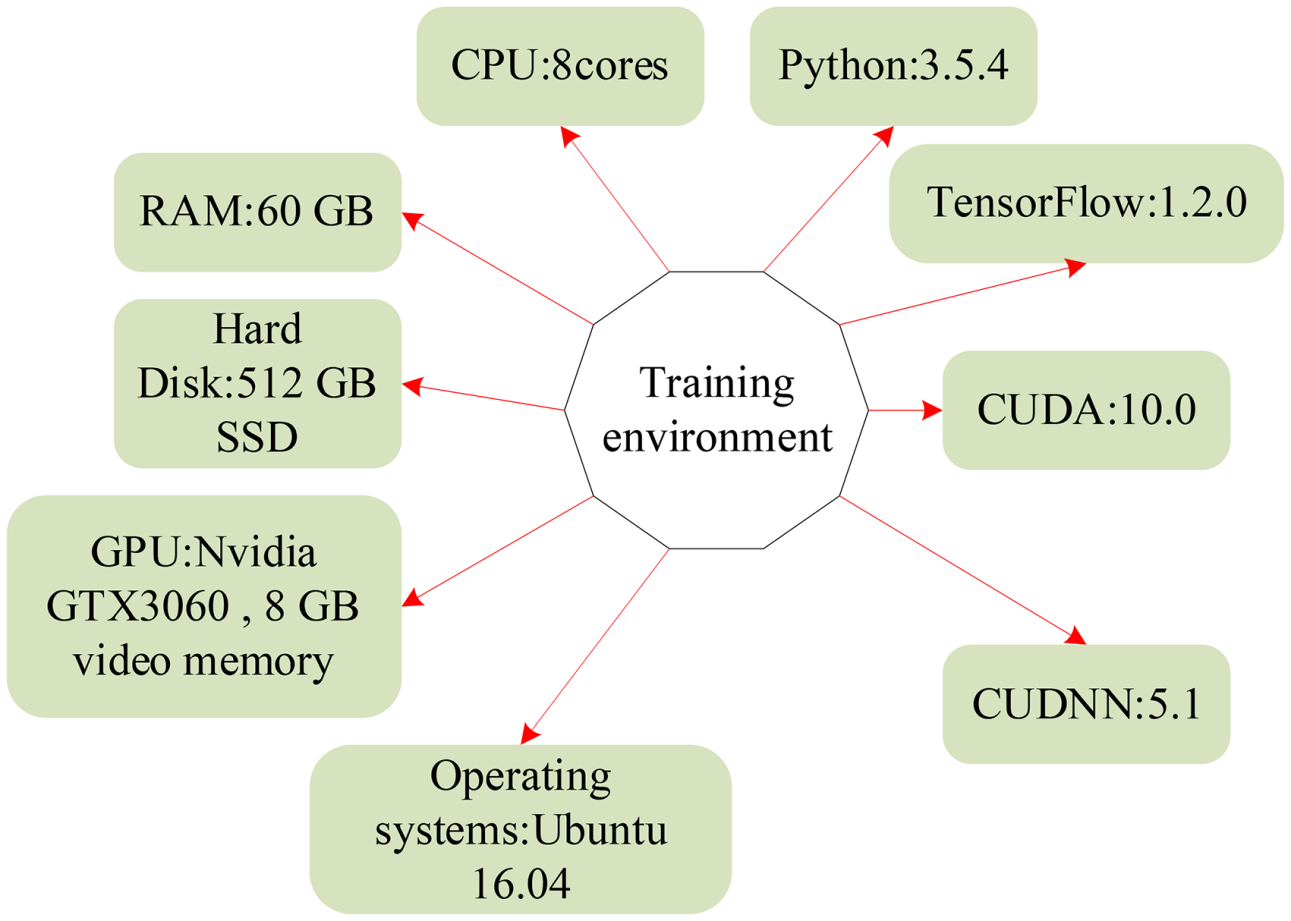
Framework diagram of the training environment.

The platform used for training data is shown in Figure 12, and the operating system is Ubuntu.

**Figure 13:**
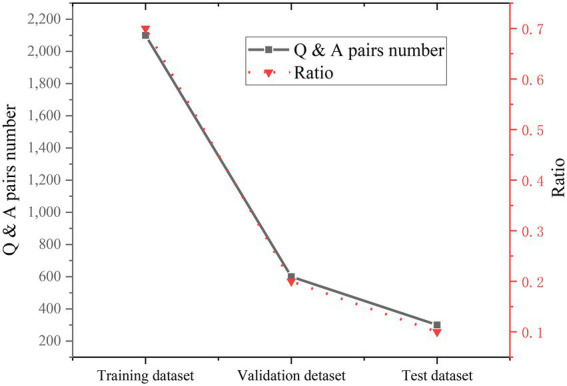
Example image of the questions.

At present, the Q&A database in the field of ceramic art on the Internet is very fragmented. Several ceramic art question bank websites are selected to crawl and clean the captured data. Finally, about 300 multiple-choice questions are selected as training data. First, visit the target page to obtain the page data, and then process and extract the required data, mainly extracting multiple choice questions. And it should be noted that the answer to the question is not given directly, so it is necessary to jump to another page to obtain the question with the answer option, as shown in Figure 13, and finally, the grabbed data are saved to a txt file.

**Figure 14:**
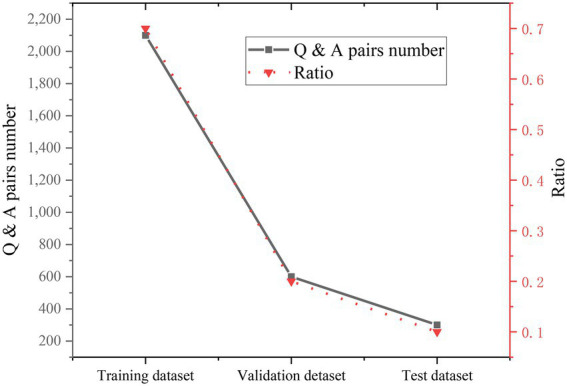
Data set allocation.

After the captured multiple-choice questions are processed to a certain extent, the data set is divided into 70% training data set, 20% verification data set, and 10% test data set. The specific data are shown in Figure 14.

### Model establishment

**Figure 15:**
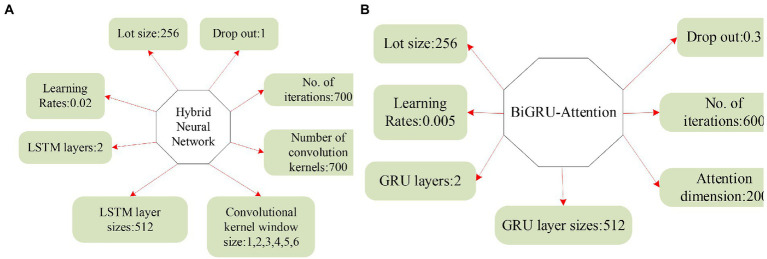
Parameters and acquisition process of two NNs. **(A)** HNN parameters. **(B)** BiGRU-Attention parameters.

The parameters of the CNN-based hybrid model and the BiGRU-Attention model are demonstrated in Figure 15.

### Objective function and evaluation index

The objective function of the model is as follows. $a^{+}$ represents positive example answer vector; $a^{-}$ represents negative example answer vector; andm represents threshold parameter.


(44) $$L=\max\left(0,m-\cos\left({q,a}^{+}\right)+\cos\left(q,a^{-}\right)\right)$$

The evaluation metrics used are Accuracy (Acc) and Mean Reciprocal Rank (MRR). Acc is the ratio of the correct number of samples to the total number of samples. MRR is the effect index of the general search algorithm, as displayed in Equations (45) and (46).


(45) $$\mathrm{Accuracy}=\frac{\mathrm{Number of samples}\;\mathrm{wit}h\;\mathrm{correct answers}}{\mathrm{Total number of samples tested}}$$

(46) $$MRR=\frac{1}{\left|Q\right|}\overset{\left|Q\right|}{\underset{i=1}{\sum }}\frac{1}{\mathrm{ran}k_{i}}$$

Q: the set of sample queries; |Q|: the number of queries; $\mathrm{ran}k_{i}$: ranking of the first correct answer of the i-th quert.

Due to some particularities of the adaptive DL algorithm of ceramic art, the traditional classification accuracy cannot be used for evaluation, so two indicators, F1 score and Area Under Curve (AUC), are introduced. The confusion matrix is shown in Table 1.

**Table 1:** Confusion matrix.

	Real category	
Forecast category	BP	EP
	BN	EN

As shown in Table 1, when the true-positive (TP) BP model predicts positive, the true label is also positive. When the true-negative (TN) EN model predicts negative, the true label is also negative. The false-positive (FP) EP model predicts positive, and the true label is negative. The false-negative (FN) BN model predicts negative, and true labels are positive.

The F1 score is an important indicator to measure the effect of the binary classification model in statistics, which can be regarded as the harmonic average of the precision rate and recall rate of the model. Its maximum value is 1 and its minimum value is 0. According to the confusion matrix in Table 1, the corresponding expressions can be obtained, as expressed in Equations (47)–(49).


(47) $$P=\frac{BP}{BP+EP}$$

(48) $$H=\frac{BP}{BP+BN}$$

(49) $$F_{1}=\frac{2\cdot P\cdot H}{P+H}$$

*P* stands for precision and *H* refers to recall.

In addition, AUC is a vital indicator to measure the quality of the two-class model, and it is the area under the Receiver Operating Characteristics (ROC) curve. It indicates the probability that positive examples are ranked ahead of negative examples. Its maximum value is 1 and its minimum value is 0. Its measurement standard is shown in Table 2.

**Table 2:** Metrics for AUC.

Range	Significance
AUC = 1	Perfect classification
1 > AUC > 0.5	Good predictive power
AUC = 0.5	No predictive power
0.5 > AUC ≥ 0	It is better to guess at random

The closer the AUC value is to 1, the better and more perfect the classification effect is, and the closer the AUC value is to 0, the worse the classification effect. The specific calculation method is indicated in Equation (50).


(50) $$AUC=\frac{\sum i\in \mathrm{positiveClassran}k_{i}-\frac{C\left(1+C\right)}{2}}{C\cdot D}$$

*C* stands for the number of positive samples, and *D* refers to the number of negative samples.

### Comparative analysis of experimental results of traditional retrieval methods

**Figure 16:**
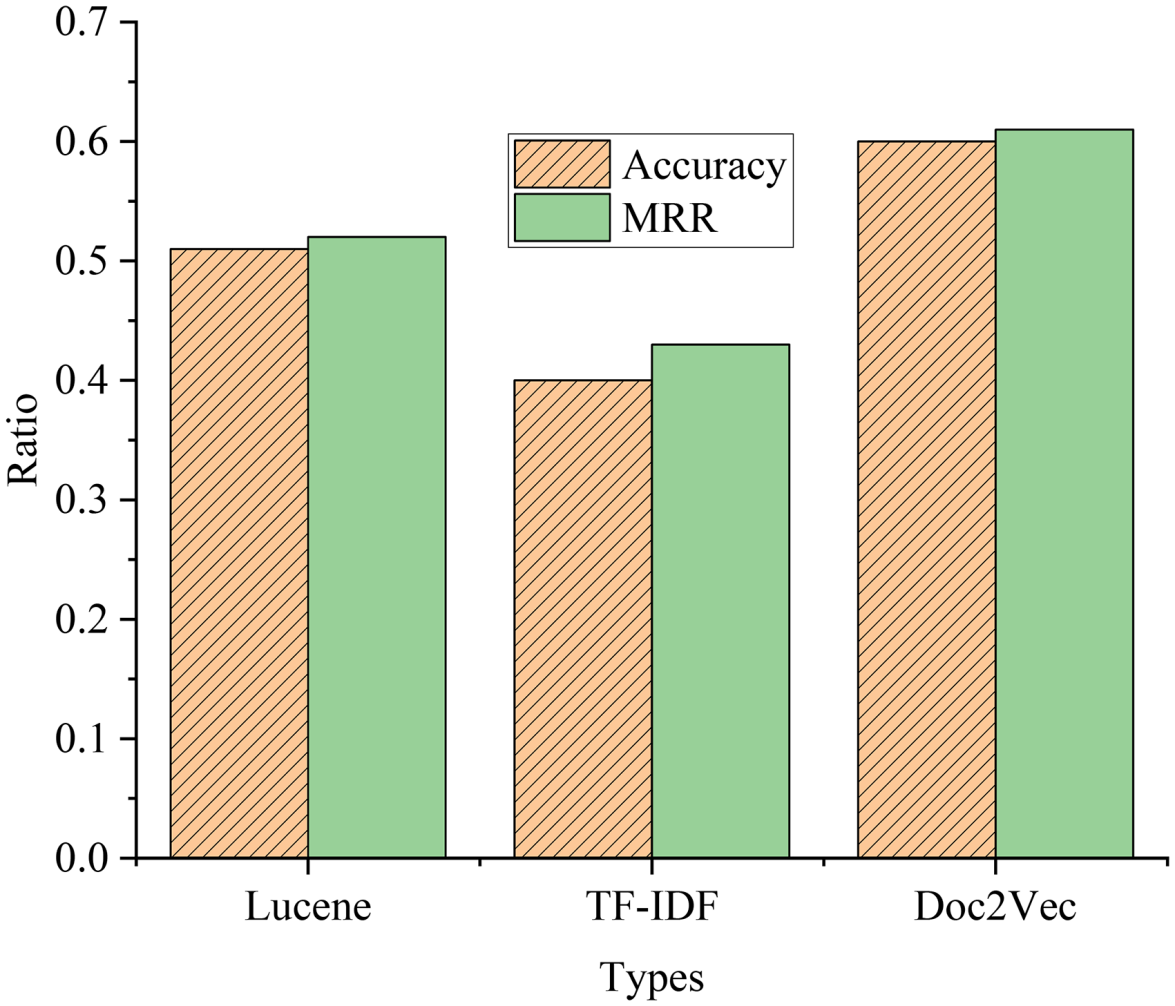
Comparison of experimental results of question-and-answer search with traditional retrieval methods.

The comparison results of the question-and-answer search experiment using the traditional retrieval system method are shown in Figure 16.

Lucene is an open-source full-text search engine toolkit that provides a complete query engine, indexing engine, and partial text analysis engine (English and German in two Western languages). TF-IDF (Term Frequency–Inverse Document Frequency) is a statistical method to assess the importance of a word to a document set or a document in a corpus. Doc2Vec is a vectorized representation of the created document. As shown in Figure 15, among the three retrieval methods, the Lucene engine is based on keyword search, with an accuracy rate of 0.51 and an MRR of 0.52; the accuracy rate of the TF-IDF method is only 0.4, and the MRR is only 0.43; the accuracy rate of the Doc2Vec method is 0.6, and the MRR is 0.61. The principle of the Lucene engine is similar to that of the TF-IDF method, and the search effect is general. Doc2Vec mainly converts words into word vectors, and the search effect is the best compared to the other two methods.

### Comparative analysis of experimental effects of basic neural network

**Figure 17:**
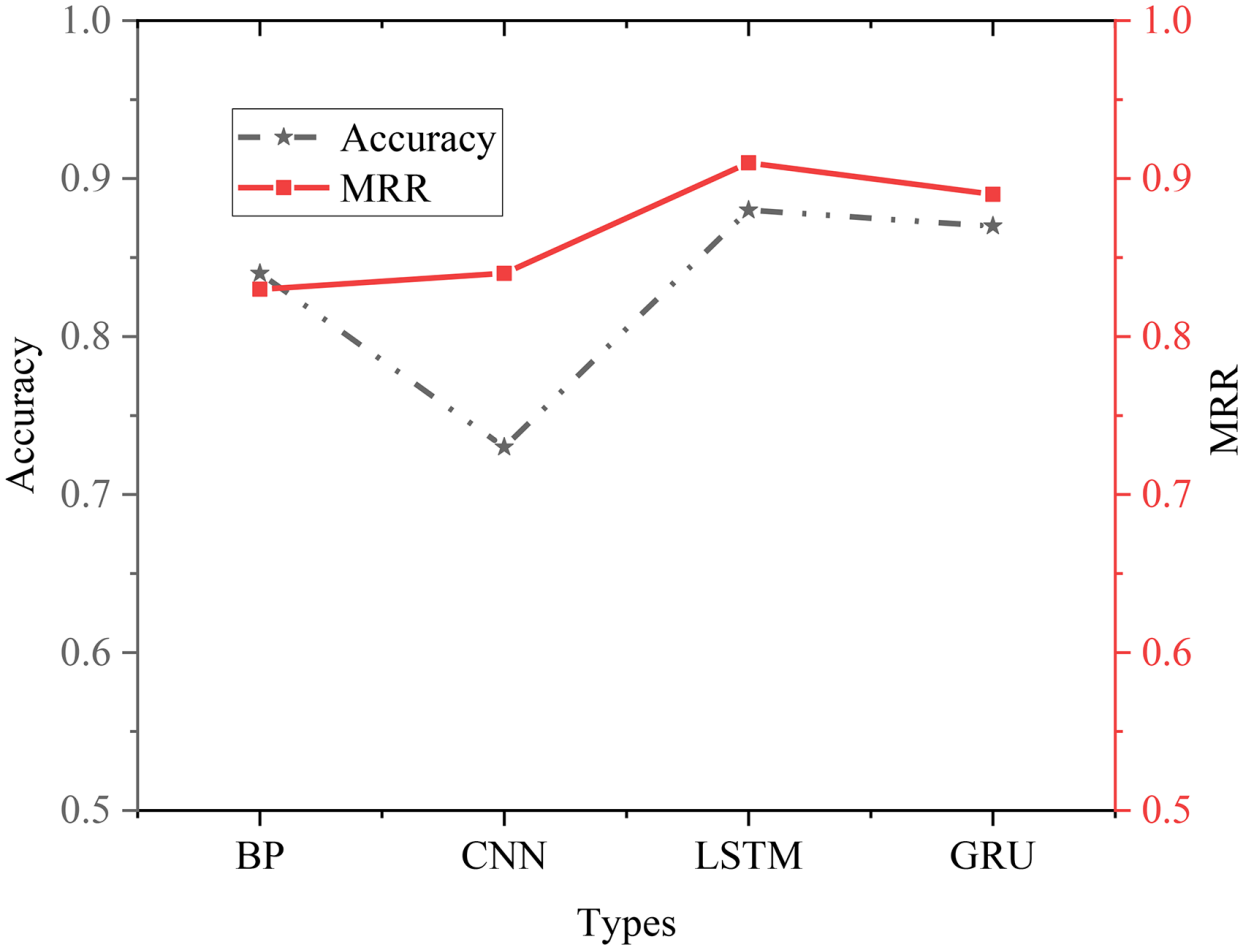
Comparison of experimental results of basic NN.

The experimental results of the basic NN are shown in Figure 17.

To better compare the performance of different models, this experiment also conducts an experimental comparative analysis of the basic NN model. It can be seen from the experimental results that the Acc of BPNN is 0.84, and the MRR is 0.83. Compared with the traditional method, the performance of BPNN is improved. Compared with the BPNN model, the performance of the CNN model has declined, and both LSTM and GRU are better than the CNN model in processing text tasks. Compared with the LSTM model, the GRU model has a simpler structure, fewer parameters, and faster training speed. The LSTM model has the best effect, but the structure is more complex than the GRU model, with more parameters and slower training speed. Therefore, when choosing a NN model, it should be selected according to the actual situation.

### Experimental comparison and analysis of LSTM-CNN model and BiGRU-Attention model

**Figure 18:**
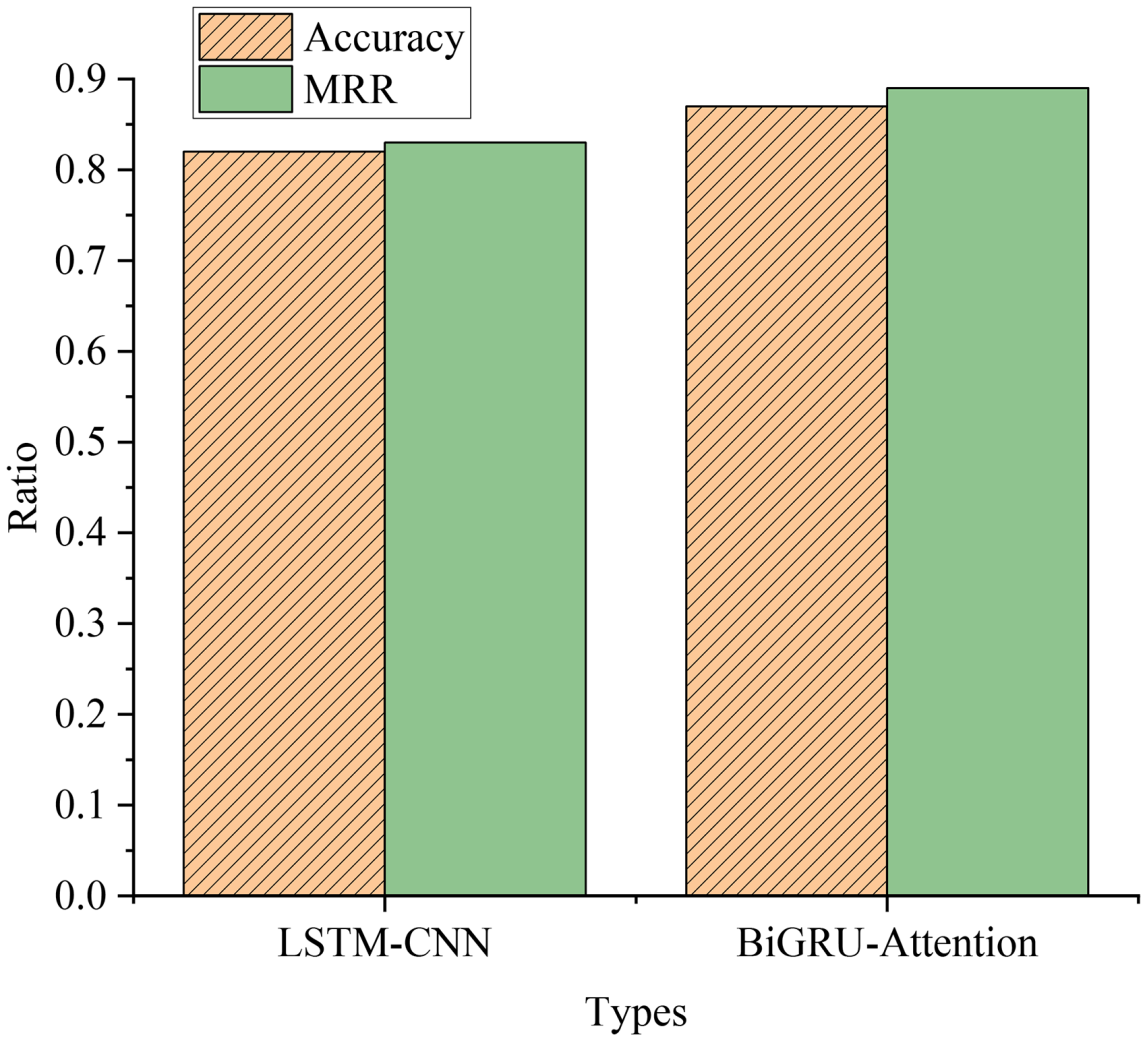
A comparison of the experimental results of the models.

Figure 18 signifies the comparative analysis of the experimental results of the LSTM-CNN model and the BiGRU-Attention model.

**Figure 19:**
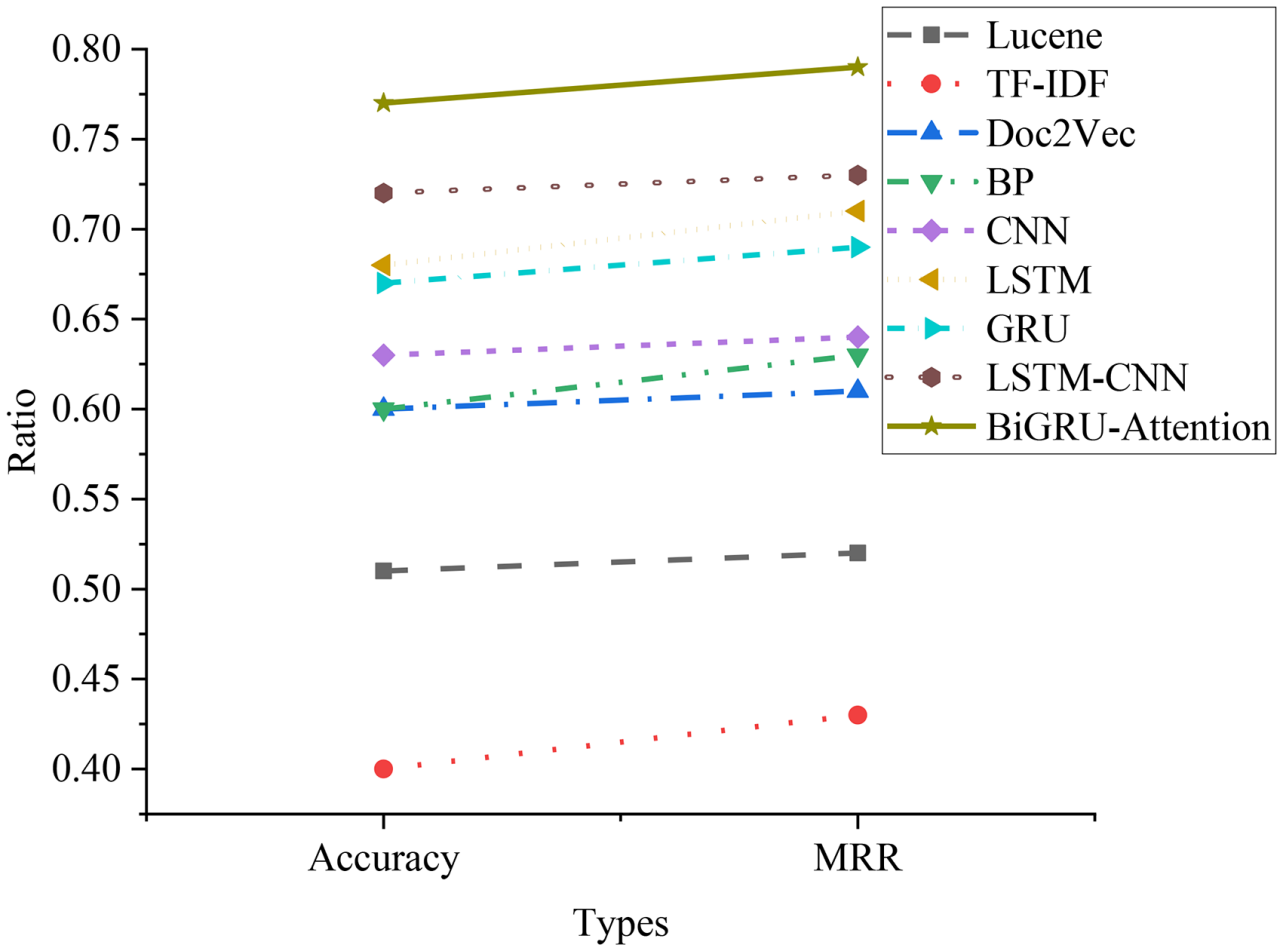
Comparison of experimental results of all models.

As shown in Figure 18, compared with the traditional model and the NN model, the used LSTM-CNN and BiGRU-Attention models are significantly better. The Acc of the LSTM-CNN model is 0.82, and the MRR is 0.83. Compared with the traditional model and the basic NN model, the Acc and MRR are much improved, and better results can be achieved. The Acc of the BiGRU-Attention model is 0.87, and the MRR is 0.89. Compared with the LSTM+CNN model, the Acc and MRR are also improved. The attention mechanism can retain more effective features after the feature weights of each step are calculated, which greatly improves the model effect. The appropriate combination of different models and the advantages of different models can effectively improve the overall effect. The comprehensive effect of all models is compared in Figure 19.

It can be clearly seen from Figure 19 that the effect of the traditional method is obviously not as good as that of the basic NN model. The main reason is that the structure of Deep Neural Networks (DNN) is superior to traditional methods, and NN can better describe and extract semantic features when processing sentence information. RNN can also retain contextual information during processing, which can represent sentences more accurately. The LSTM-CNN model and the BiGRU-Attention model are better than the basic NN model. After the CNN model is combined with the LSTM model, the sentence features can be better extracted. Even in the face of complex sentences, the HNN model still works well. BiGRU can obtain more time series information. Combined with contextual semantic features and attention mechanisms, it can retain more effective features during calculation, so that the model works well when dealing with long sequences.

**Figure 20:**
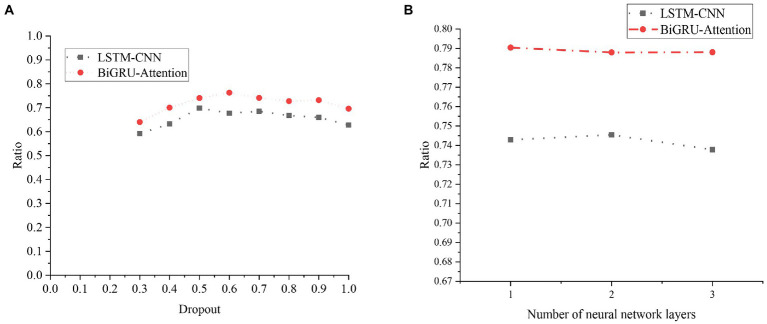
Experimental results of the LSTM model and the BiGRU-Attention model with different parameters. **(A)** Experimental results of different Dropout parameters. **(B)** Experimental results of different NN layers.

For the two models, the comparison chart of different Dropout parameters and different numbers of NN layer models is shown in Figure 20.

Figure 20A indicates that setting different Dropouts has a certain impact on the model effect. When the Dropout is 0.5, the LSTM-CNN model has the best effect, and when the Dropout is greater than 0.5, the effect of the LSTM-CNN model begins to decline. The BiGRU-Attention model has the best effect when the Dropout is 0.6, and the effect decreases when the Dropout is greater than 0.6. Figure 20B reveals that the number of layers of the NN is selected as 1, 2, and 3 for the experiment. The effects of the two models have not changed much, and the increase in the number of layers will not significantly affect the experimental results. The increase in the number of NN layers will also slow down the training speed, so finally, the number of NN layers is 1.

## Conclusion

Based on the automatic QA system established by DL, new teaching strategies are analyzed. The Internet is combined with the automatic QA system to help students solve problems encountered in the process of learning. Firstly, the advantages and disadvantages of BPNN, CNN, LSTM, and GRU are analyzed one by one. Secondly, models are constructed using different NNs. The LSTM is combined with the CNN to implement the LSTM-CNN model, which is compared and analyzed with the BiGRU-Attention model. Finally, experiments manifest that the accuracy and MRR of traditional retrieval methods can only reach about 0.5 on average. In the basic NN, BPNN, CNN, LSTM, and GRU, the average Acc of the BPNN model and the CNN model is about 0.81, and the average MRR is about 0.84. The average Acc of the LSTM model and the GRU model is about 0.82, and the average MRR is 0.83, which is obviously better. After the CNN model is combined with the LSTM model, the sentence features can be better extracted. Even in the face of complex sentences, the HNN model still works well. BiGRU can obtain more time series information. Combined with contextual semantic features and attention mechanism, it can retain more effective features during calculation, so that the model works well when dealing with long sequences. The DL-based automatic QA system not only helps teachers explore new teaching strategies, but also assists students customize personalized learning plans to improve students’ learning effects. However, the data of the Chinese QA system is still very small. For the DL model, the more data in the sample database, the more accurate the experimental results. Moreover, after solving the problem of individualization of students, the improvement of learning effect and the exercises between course content and teaching strategies still need further practice tests. In the future, research in this area will continue to improve the experiment to achieve better results.

## Data availability statement

The raw data supporting the conclusions of this article will be made available by the author, without undue reservation.

## Ethics statement

The studies involving human participants were reviewed and approved by Xinyang Normal University Ethics Committee. The patients/participants provided their written informed consent to participate in this study. Written informed consent was obtained from the individual(s) for the publication of any potentially identifiable images or data included in this article.

## Author contributions

All authors listed have made a substantial, direct, and intellectual contribution to the work, and approved it for publication.

## Funding

This work was supported by the 2022 National Art Fund Youth Artistic Creation Talent Project: “Praise to Dabie Mountain” pottery creations series [2022-A-06-(129)-598] and the Henan Province Philosophy and Social Science Planning Project: “Research on Pottery Unearthed from the Chu Tomb of Warring-States Age Nobles at Yangcheng Site in Xinyang, Henan” (2021BYS041).

## Conflict of interest

The authors declare that the research was conducted in the absence of any commercial or financial relationships that could be construed as a potential conflict of interest.

## Publisher’s note

All claims expressed in this article are solely those of the authors and do not necessarily represent those of their affiliated organizations, or those of the publisher, the editors and the reviewers. Any product that may be evaluated in this article, or claim that may be made by its manufacturer, is not guaranteed or endorsed by the publisher. The author confirms being the sole contributor of this work and has approved it for publication.
